# Supplementary material for: Silicone engineered anisotropic lithography for ultrahigh-density OLEDs
Source: Nat Commun. 2022 Dec 12;13:6775. doi: 10.1038/s41467-022-34531-y (PMC9744739; doi:10.1038/s41467-022-34531-y)
Supplement: Supplementary file 1 — Supplementary Information [file 41467_2022_34531_MOESM1_ESM.pdf]

Supplementary Information

Silicone engineered anisotropic lithography for ultrahigh-density OLEDs

Hyukmin Kweon[1]†, Keun-Yeong Choi[2]†, Han Wool Park[1]†, Ryungyu Lee[2], Ukjin Jeong[1], Min Jung Kim[3], Hyunmin Hong[3], Borina Ha[1], Sein Lee[4], Jang-Yeon Kwon[4], Kwun-Bum Chung[3], Moon Sung Kang[5,6], Hojin Lee[2,7]*, Do Hwan Kim[1,8]*

[1]Department of Chemical Engineering, Hanyang University, Seoul 04763, Republic of Korea.

[2]School of Information Communication Convergence Technology, Soongsil University, Seoul 06978, Republic of Korea.

[3]Division of Physics and Semiconductor Science, Dongguk University, Seoul 04620, Republic of Korea.

[4]School of Integrated Technology, Yonsei University, Incheon 21983, Republic of Korea.

[5]Department of Chemical and Biomolecular Engineering, Sogang University, Seoul 04107, Republic of Korea.

[6]Institute of Emergent Materials, Sogang University, Seoul 04107, Republic of Korea.

[7]School of Electronic Engineering, Soongsil University, Seoul 06978, Republic of Korea.

[8]Institute of Nano Science and Technology, Hanyang University, Seoul 04763, Republic of Korea.

†These authors contributed equally to this work.

*Corresponding author. E-mail: hojinl@ssu.ac.kr (H.L.); dhkim76@hanyang.ac.kr (D.H.K.)

This PDF file includes:

Supplementary Notes 1 to 2
Supplementary Figures 1 to 24
Supplementary Tables 1 to 2
Supplementary References 1 to 23

Separate supplementary file for this manuscript: Supplementary Movie 1

Supplementary Note 1 | A role of silicone (Si-O-Si) network of the SI-OLES for reactive ion etching (RIE)-coupled photolithography

In general, organic light-emitting semiconductor (OLES) has been considered not acceptable to reactive ion etching (RIE)-based photolithography because of the two major drawbacks as follow; i) the OLES films can be highly vulnerable to solutions (e.g., photoresist, developer, and stripper) employed in photolithography process due to intrinsically their low chemical robustness[1,2], and ii) alkyl-based chemical structure of the OLES is easily broken by reactive etching gases (typically oxygen or fluorine-based gases) and ion bombardment during the RIE process, so that damage on its π-conjugation core and severe isotropic etching profiles could be accompanied[3,4].

$$\text{-CH}_2\text{-} + 2\text{O}^* \rightarrow \text{CO} + \text{H}_2\text{O or CO}_2 + \text{H}_2 \text{(Volatile reactants)} \quad (1)$$

$$\text{-CH}_2\text{-} + 6\text{F}^* \rightarrow \text{CF}_4 + 2\text{HF (Volatile reactants)} \quad (2)$$

An introduction of ladder-like silicone (Si-O-Si) network into the OLES can be an ultimate solution to solve both problems, simultaneously. The OLES molecules can be confined in the incorporated silicone network by molecular entanglement, resulting in possessing chemical tolerance (**Supplementary Fig. 4**). More importantly, the CH_2 scission reactions of the SI-OLES film are effectively alleviated in the both horizontal and vertical etching direction because silicone blocks on the surface of the film or exposed at sidewall of etched trench can form a non-volatile etch-blocking layer (EBL) (Si_xO_y or SiO_xF_y) by chemical reaction with gas radicals (O^* or F^*) during RIE process. This is an important building block to secure dry etching resistance and anisotropic etching behavior of the SI-OLES film, which is highly analogous capability to traditional silicon materials. It is noted that even for silicon (Si), unless the non-volatile EBL is not employed, isotropic etching behavior can be dominated[5,6]. This strongly indicates that development of the EBL is a key step to realize anisotropic etching mechanism of the RIE process.

Supplementary Note 2 | RIE-based etching reaction of the SI-OLES depending on etch directionality.

The RIE process is a combination of chemical and physical etching, corresponding to gas radical reaction and ion bombardment, respectively. As shown in **Figure 1**, chemical etching reaction occurs in both vertical and horizontal etching directions, on the other hand, ion

bombardment is responsible for only vertically etching. This implies that etching reaction of the SI-OLES during the RIE process could differ depending on etch directionality. Firstly, etching gases (Ar/O_2 or CF_4) possessing high electron affinity are absorbed on the surface of the SI-OLES film, so that surface oxidation is induced[7-9] (**Fig. 2c-e**). As discussed in **Supplementary Note 1**, in the case of the SI-OLES, the non-volatile EBL can be rendered in both horizontal and vertical direction, rather than chemical etching reaction (i.e., CH_2 scission) which predominantly occurs in the OLES. In the horizontal direction, the etching behavior is successfully suppressed during the RIE process because not only radical etching reaction is effectively hindered owing to the EBL, but also ion bombardment exhibits a biased etch directionality in the vertical direction. In contrast, in the vertical direction, the formed EBL is effortlessly removed by the energetic ion bombardment, so that vertical etching is facilitating by synergetic effect of the ion bombardment and chemical etching reaction. It is noted that the thickness of sidewall EBL is too tiny (a few nanometers)[10] to accurately investigate its characteristics, thus, alternatively, we analyzed the non-volatile EBL formed on the surface of the SI-OLES film by intentionally reducing strength of ion bombardment with the lowest radio frequency (RF) power to prevent destruction of the EBL in vertical direction[11] (**Fig. 2a-g**).

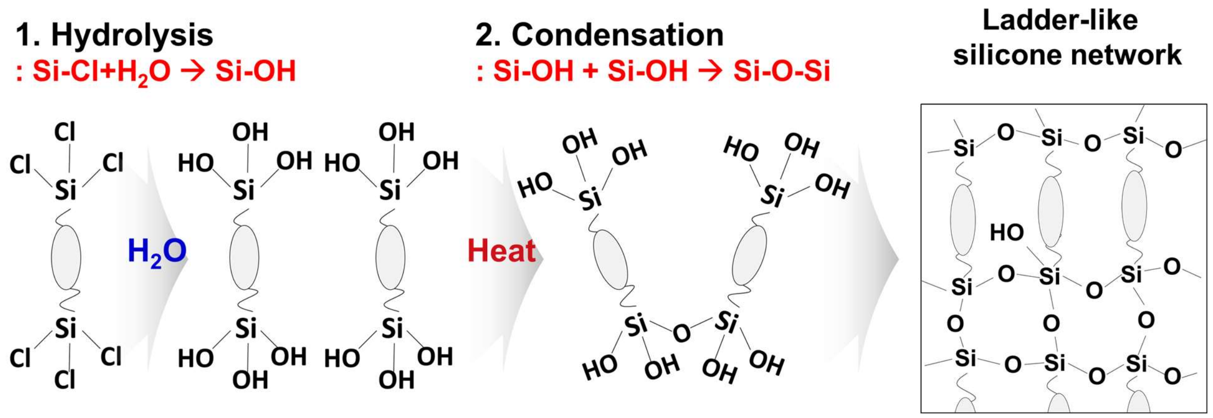

Supplementary Figure 1 | Schematic diagram showing sol-gel reactions of silicone network precursors. Through hydrolysis and condensation reactions of silicone network precursors, ladder-like silicone networks can be built in the OLES matrix.

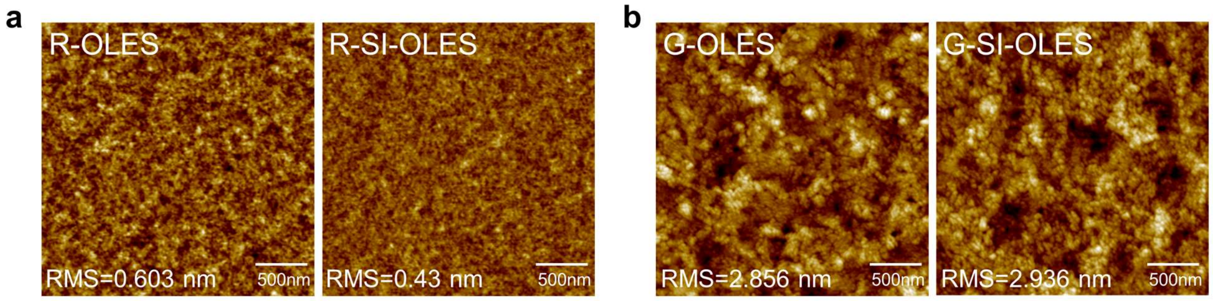

Supplementary Figure 2 | AFM images of the OLES and the SI-OLES films. Topographic images of the R-OLES/R-SI-OLES (**a**) and the G-OLES/G-SI-OLES (**b**), respectively.

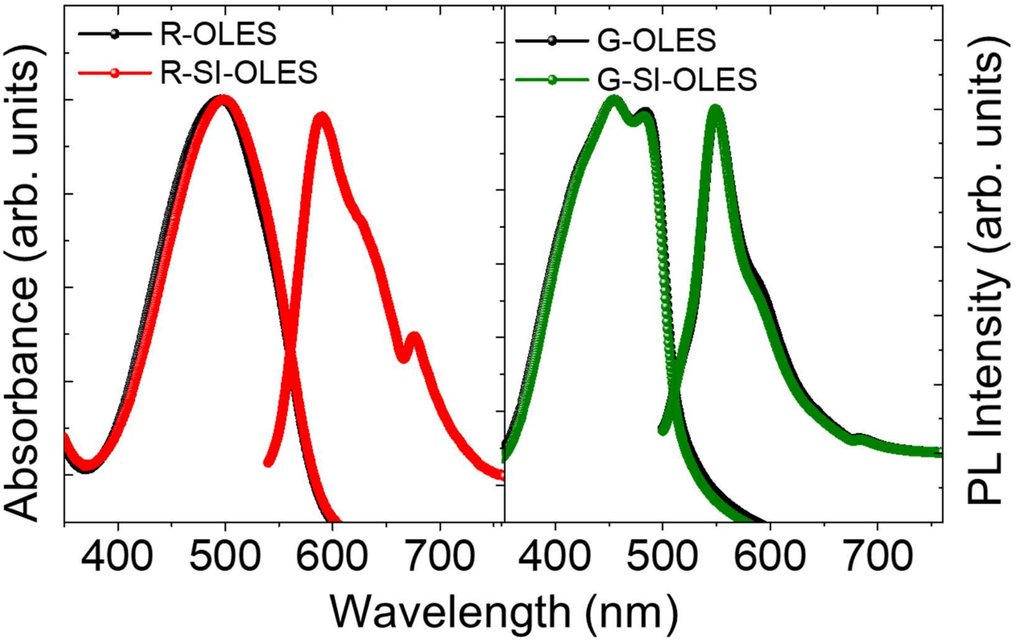

Supplementary Figure 3 | UV-vis-NIR and PL spectra of the OLES and the SI-OLES films. The PL spectra of the R-OLES and the R-SI-OLES (left) was obtained with 520 nm excitation, and that of the G-OLES and the G-SI-OLES (right) was obtained at 480 nm excitation.

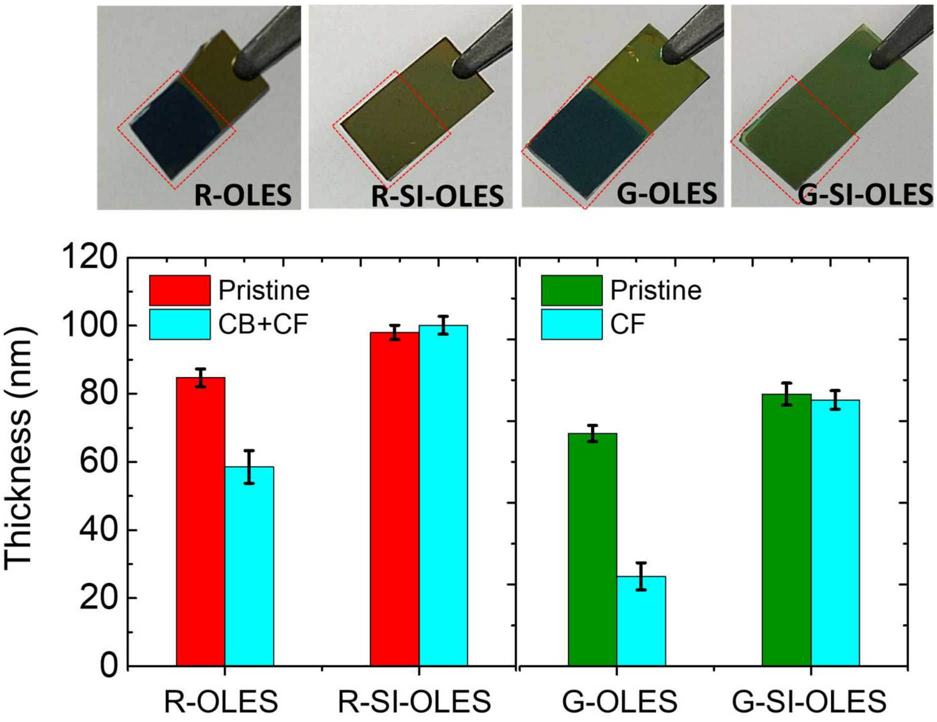

Supplementary Figure 4 | Evaluation of chemical resistance of the OLES and the SI-OLES film. Camera images and film thickness of the R-OLES/R-SI-OLES (left) and the G-OLES/G-SI-OLES (right), respectively, before and after dipping in mother solvents (CB and CF). Mean values with ± standard variations are indicated in data.

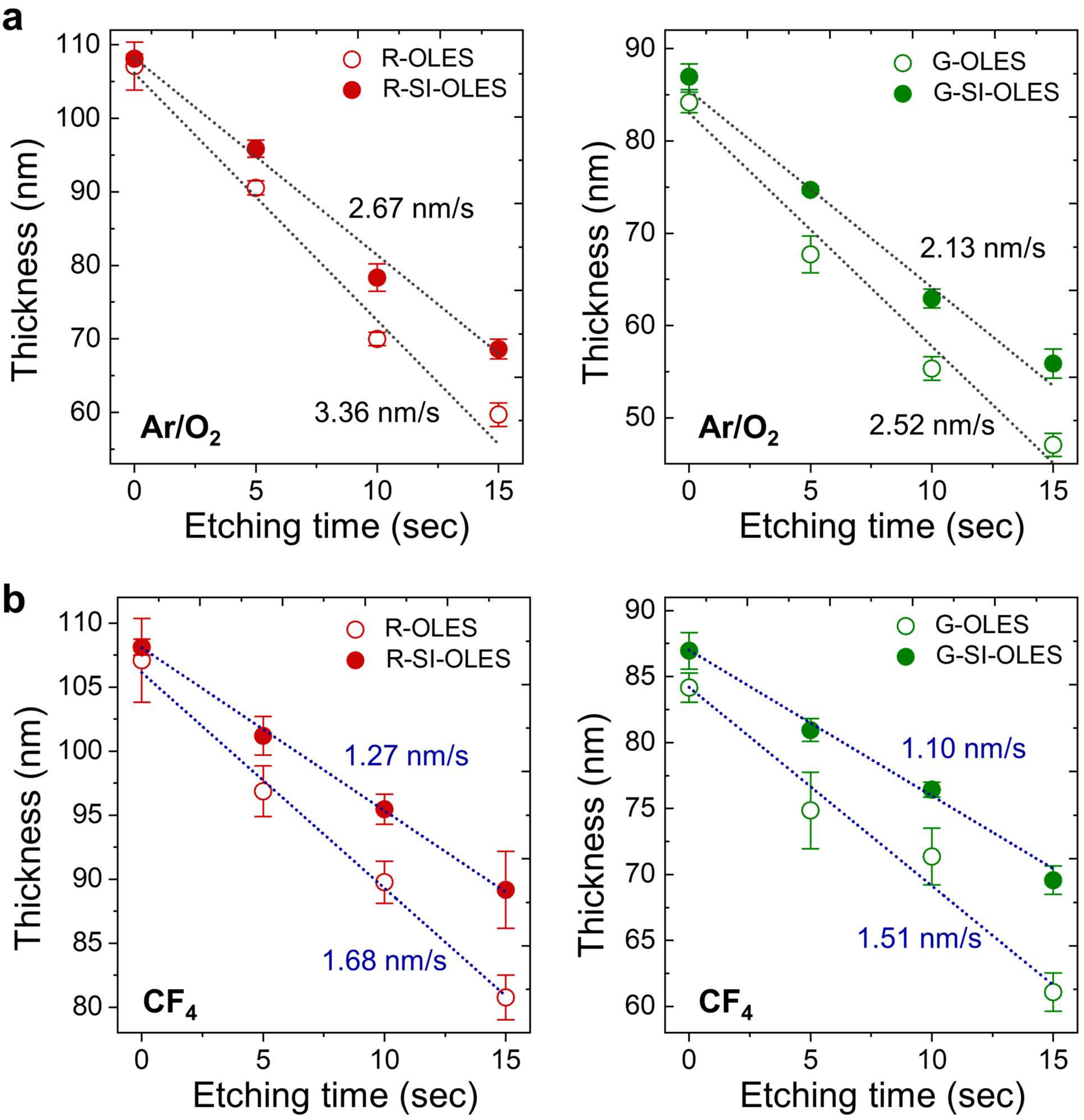

Supplementary Figure 5 | Evaluation of etching resistance of the OLES and the SI-OLES films. A variation of film thickness of the OLES and the SI-OLES films was measured as a function of the RIE etching time based on Ar/O_2 (**a**) and CF_4 gases (**b**), respectively. Mean values with ± standard variations are indicated in data. The SI-OLES films exhibited higher dry etching resistance than the OLES films.

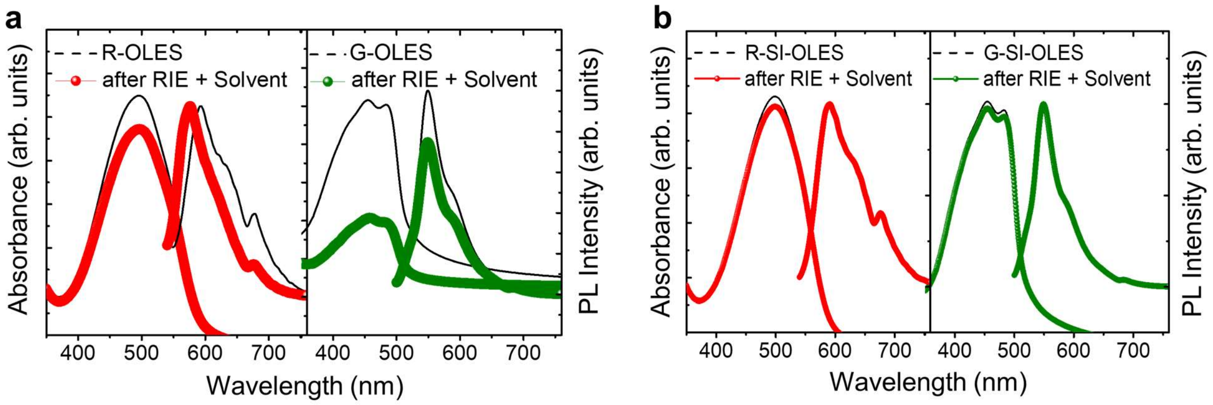

Supplementary Figure 6 | Retentive tendency of optoelectronic characteristics. Optoelectronic properties of the OLES films (**a**) and the SI-OLES films (**b**) were evaluated depending on exposure of the RIE and solvent treatment.

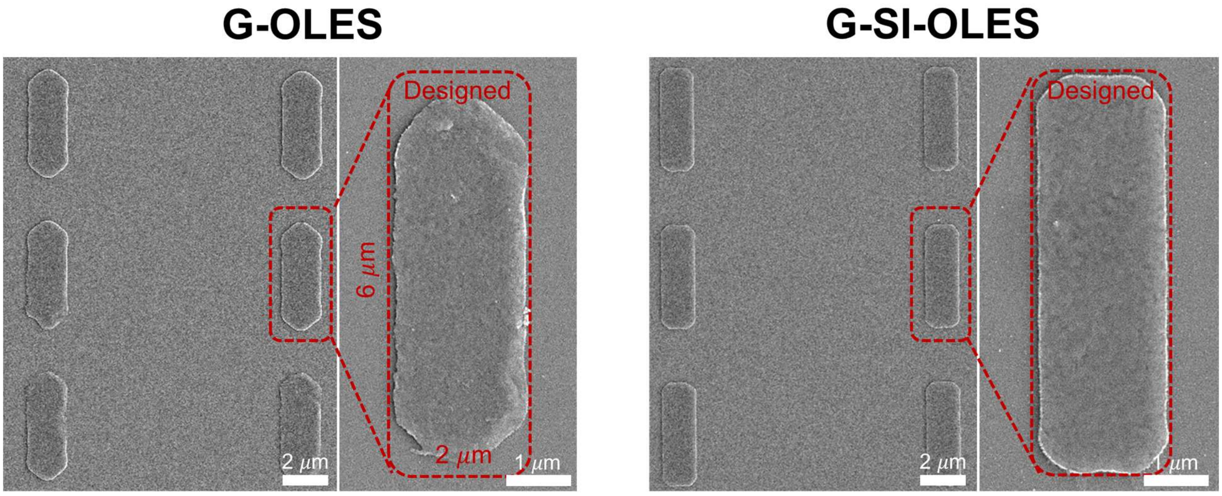

Supplementary Figure 7 | Comparison of micro-pattern property between the G-OLES and the G-SI-OLES. FE-SEM images of the both the G-OLES and the G-SI-OLES patterns designed with 2 μm x 6 μm dimension.

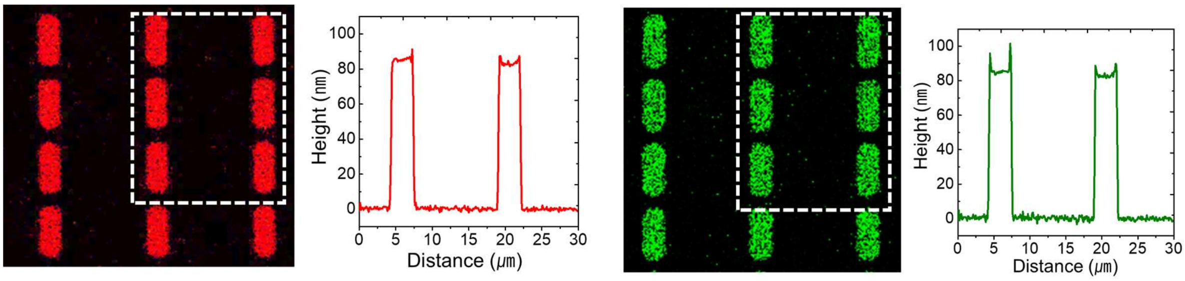

Supplementary Figure 8 | Fluorescence images of micropatterned SI-OLES films. The dimension of the micro-patterns was 2 μm x 6 μm, and both pattern arrays were fabricated by the RCP process.

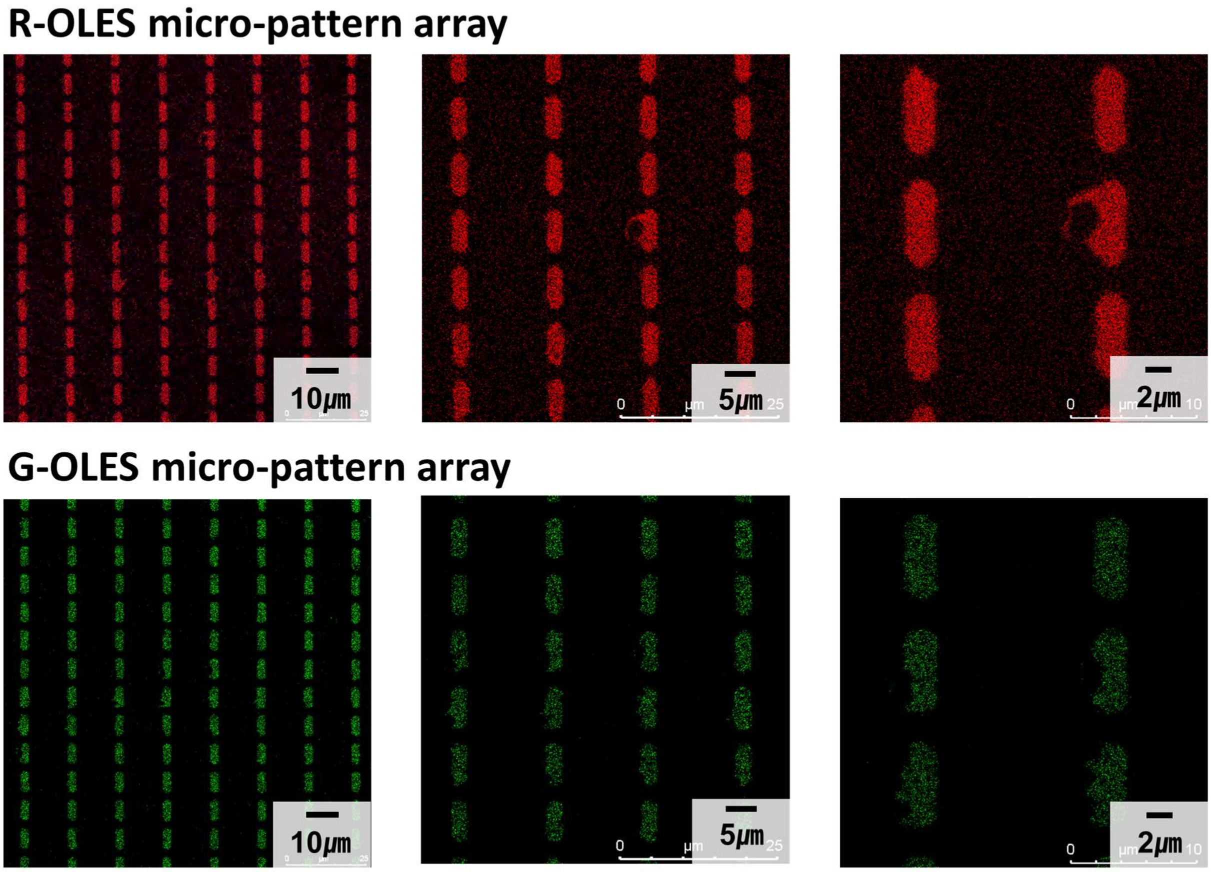

Supplementary Figure 9 | Fluorescence images of micropatterned OLES films. The dimension of the micro-patterns was 2 μm x 6 μm, and both pattern arrays were fabricated by the RCP process.

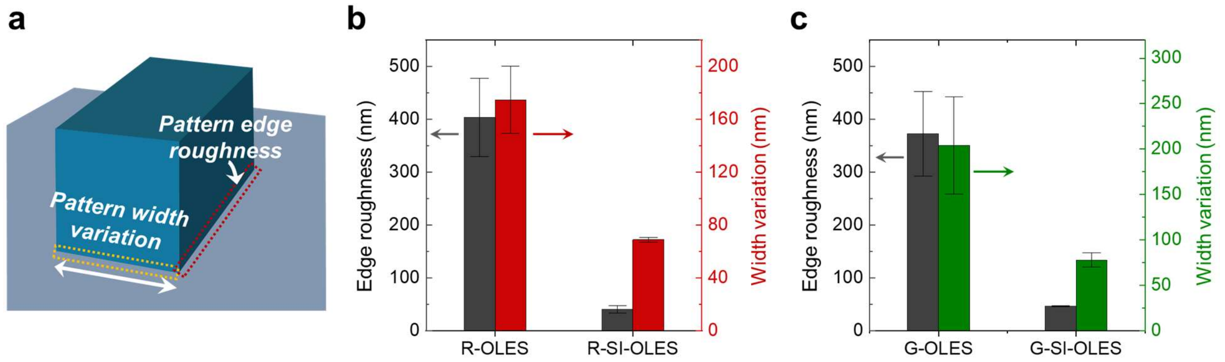

Supplementary Figure 10 | Evaluation of pattern fidelity of micropatterns. a, Conceptual illustration of pattern width variation and edge roughness. **b**,**c**, Evaluation of the key parameters of pattern fidelity corresponding to the OLES and SI-OLES-based micropatterns (2 μm × 6 μm). Mean values with ± standard variations are indicated in data.

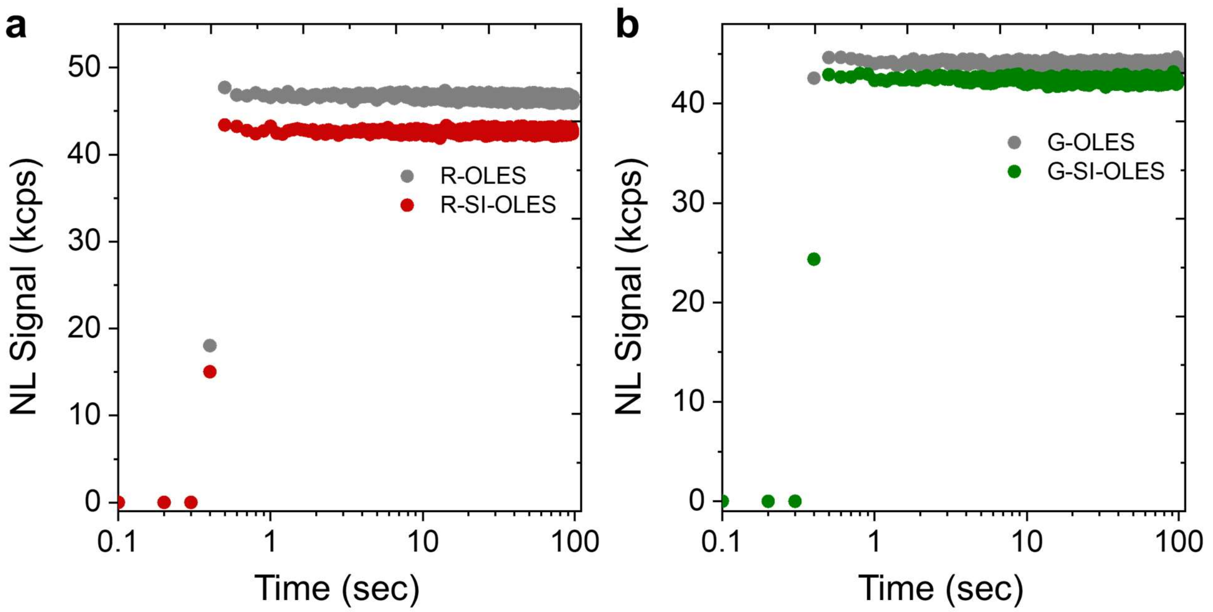

Supplementary Figure 11 | Second-harmonic generation (SHG) spectroscopy. Non-linear (NL) signal intensities of the R-OLES/R-SI-OLES (**a**) and the G-OLES/G-SI-OLES (**b**) in micro-pattern arrays, respectively.

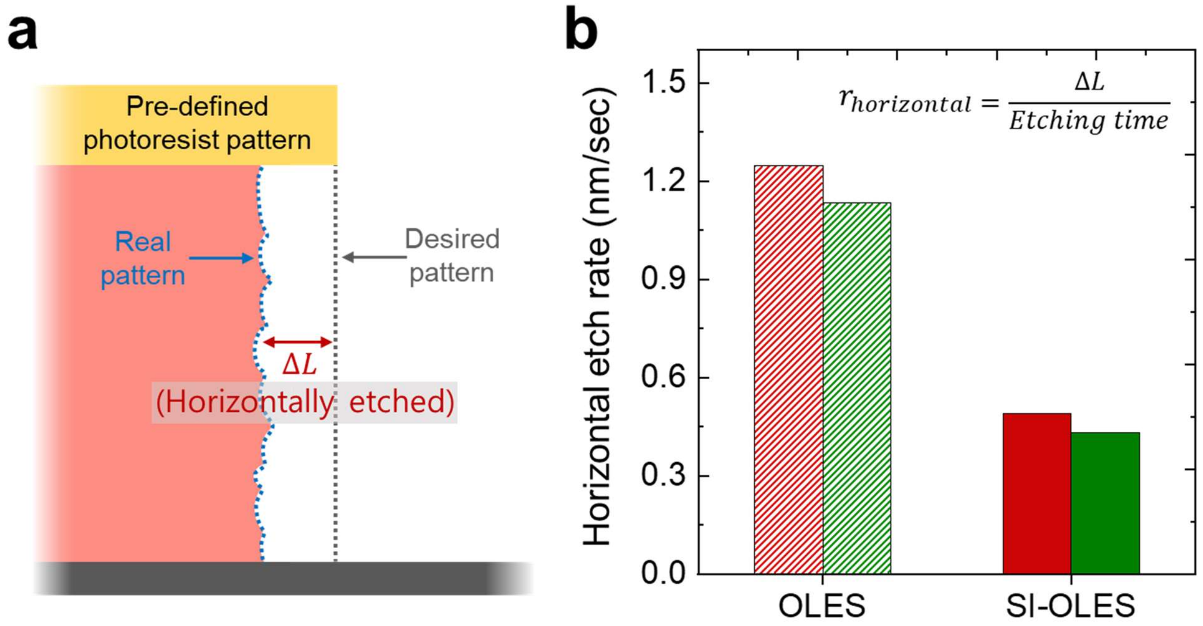

Supplementary Figure 12 | Determination of horizontal etch rate. **a**, Schematic illustration of side etching behavior induced by horizontal etching profiles. **b**, Calculation of horizontal etch rates of R- and G-OLES/SI-OLES, respectively.

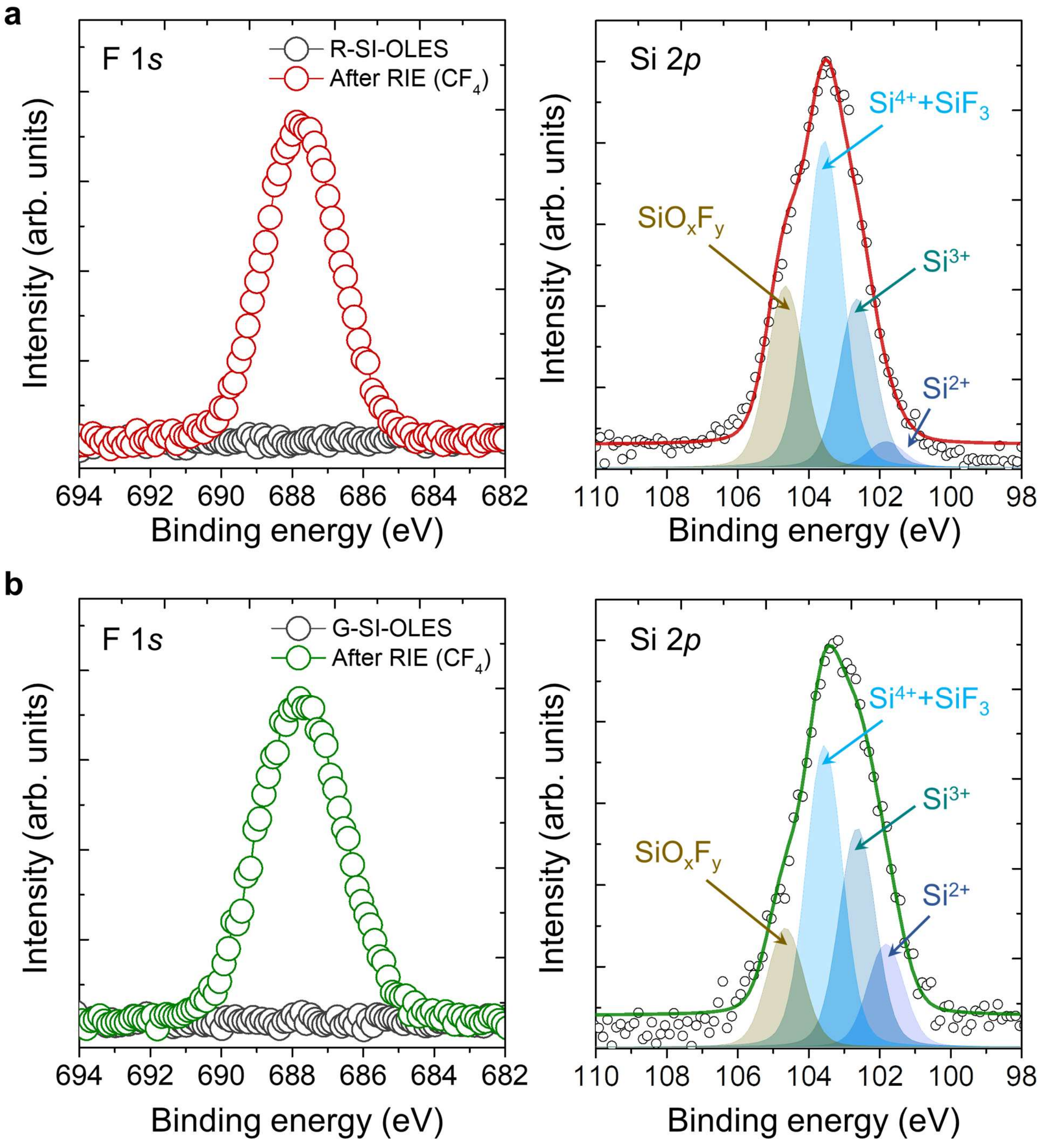

Supplementary Figure 13 | Characterization of the SI-OLES films after CF_4-based RIE process. a,b, XPS spectra of F 1*s* and Si 2*p* of the R- and G-SI-OLES, respectively[12]. The binding energy (687 eV) of F 1*s* indicates fluorine atoms bonded to SiO_x molecules[13].

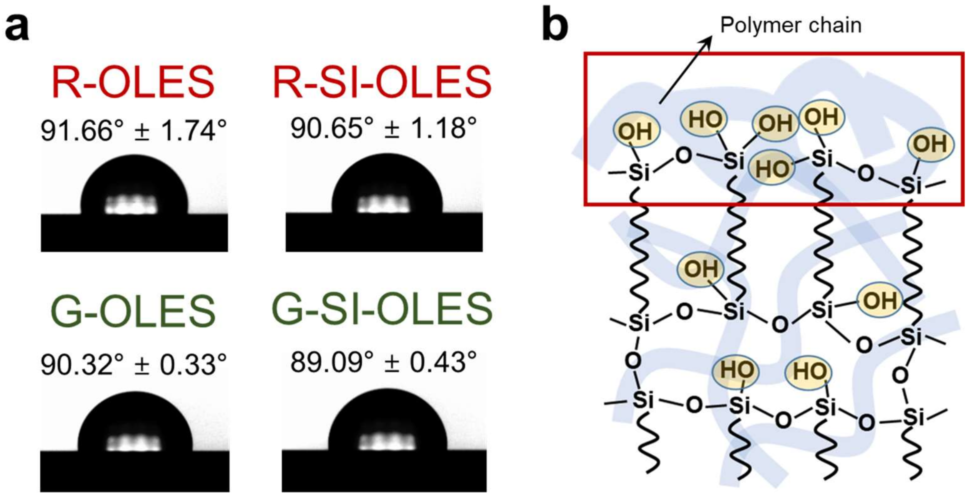

Supplementary Figure 14 | Comparison of contact angle between the OLES and the SI-OLES films. a, Contact angle of the OLES and the SI-OLES films with water droplet. Mean values with ± standard variations are indicated in data. **b**, A schematic of chemical structure of the SI-OLES film.

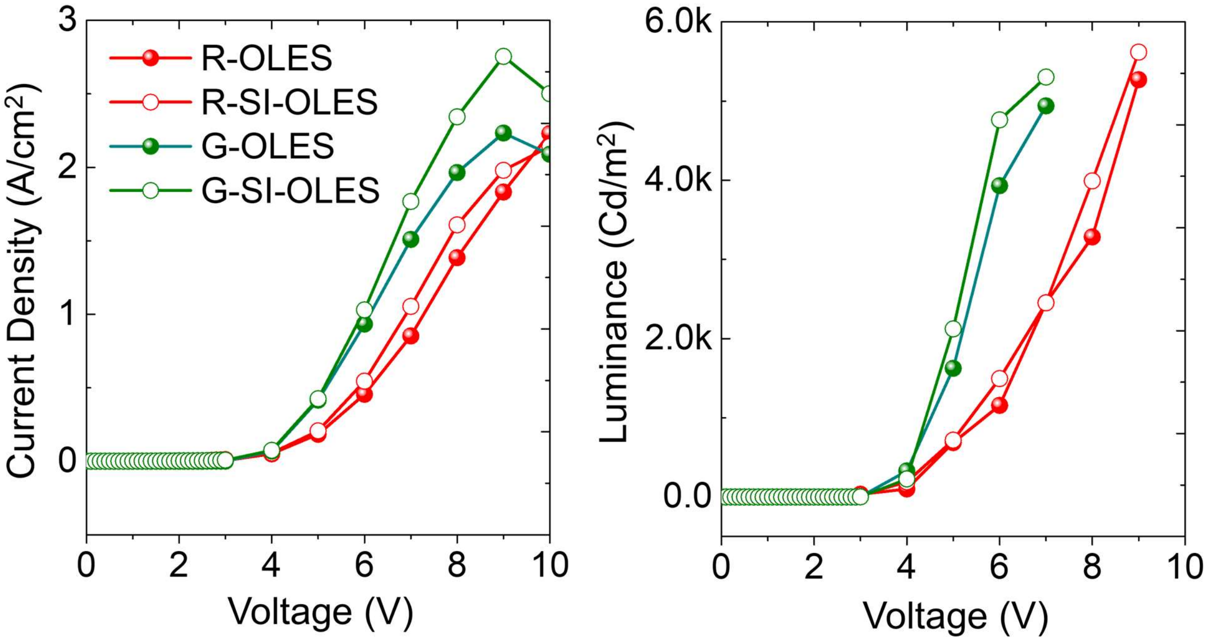

Supplementary Figure 15 | EL performance of the OLEDs based on the OLES and the SI-OLES. Current density (J)-voltage (V)-luminance(L) characteristics of the OLES and SI-OLES-based OLEDs.

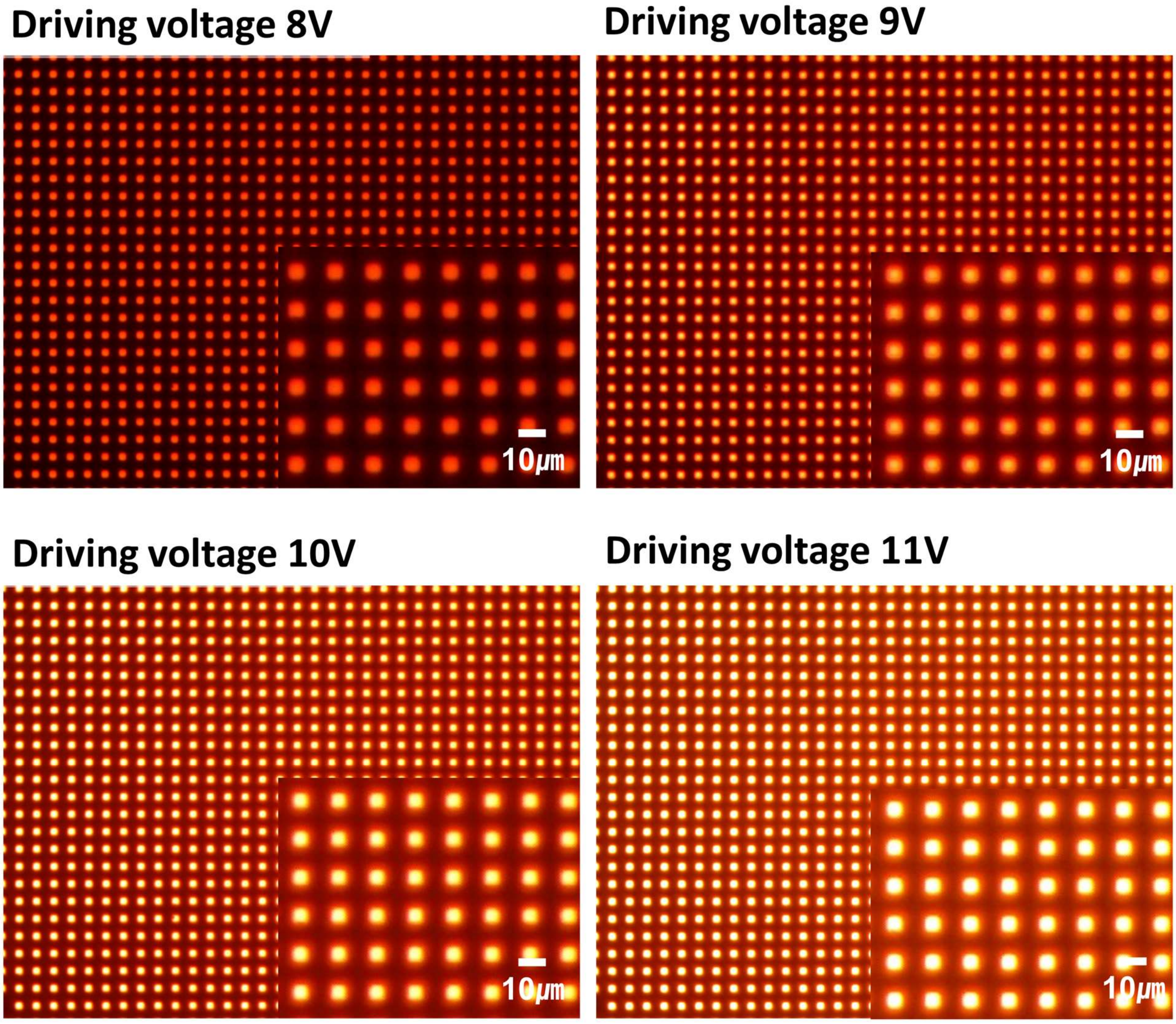

Supplementary Figure 16 | Voltage dependency of EL performance of the R-SI-OLED. Operation image of the R-SI-OLED according to voltage variation based on dot pixels with 10 μm size.

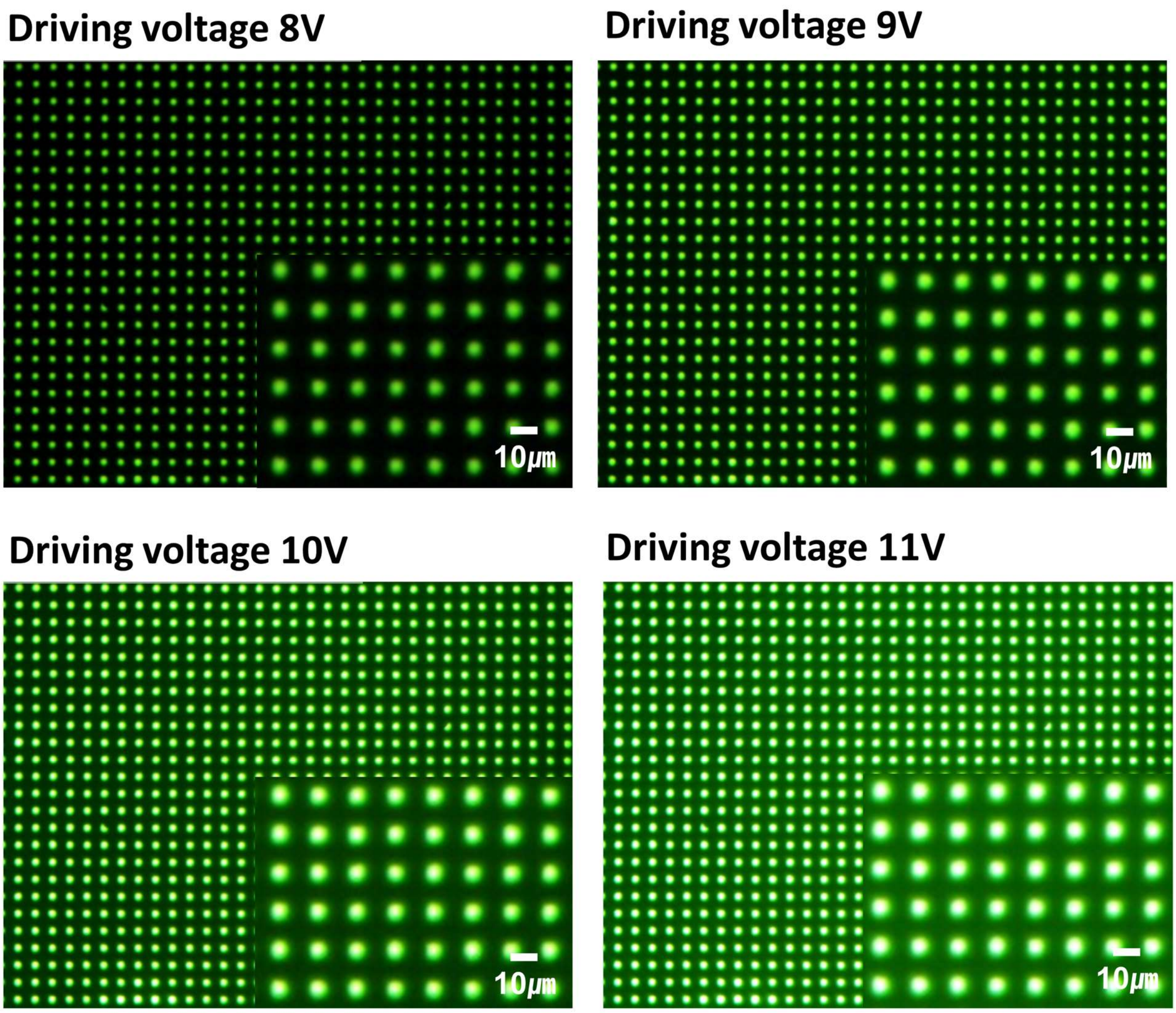

Supplementary Figure 17 | Voltage dependency of EL performance of the G-SI-OLED. Operation image of the G-SI-OLED according to voltage variation based on dot pixels with 10 μm size.

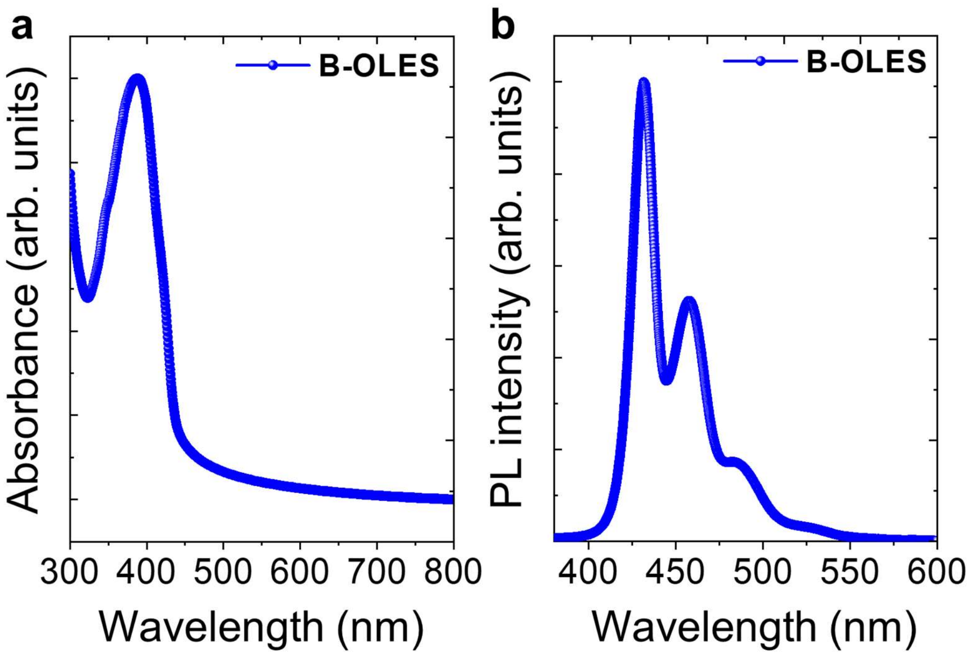

Supplementary Figure 18 | Optoelectronic property of B-OLES film. UV-vis-NIR (**a**) and PL spectra (**b**) of B-OLES.

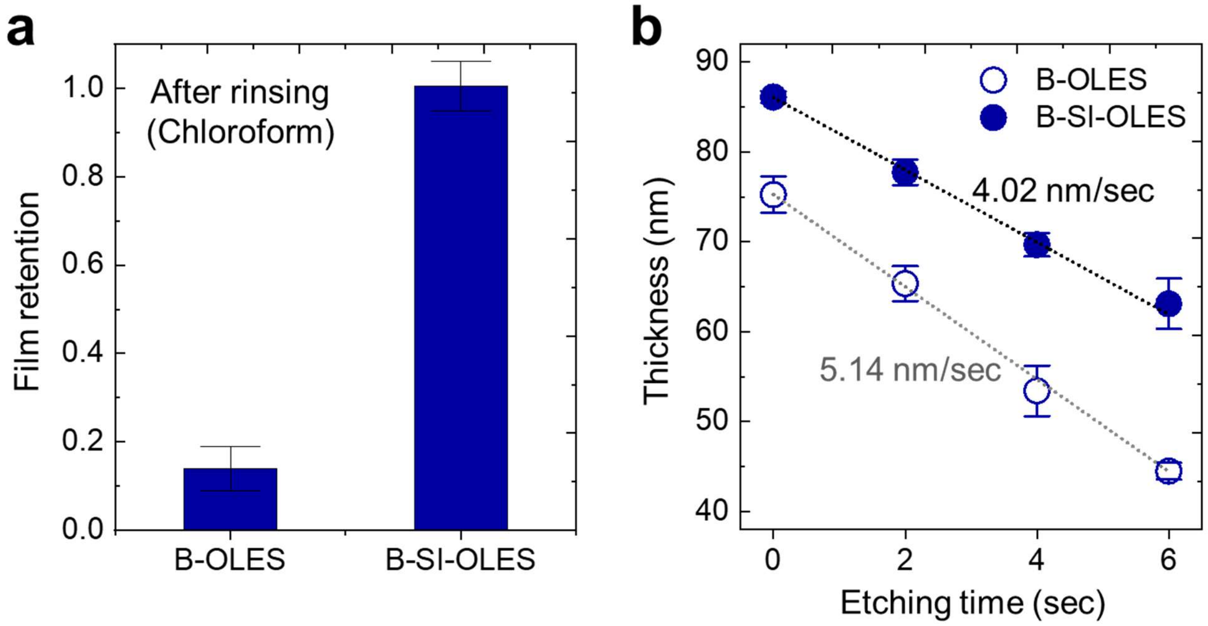

Supplementary Figure 19 | Evaluation of chemical and physical robustness of the B-SI-OLES film. a, Film retention of B-SI-OLES after solvent rinsing process. **b**, Variation of film thickness of B-OLES and B-SI-OLES films was measured as a function of the RIE etching time based on Ar/O_2. Mean values with ± standard variations are indicated in data.

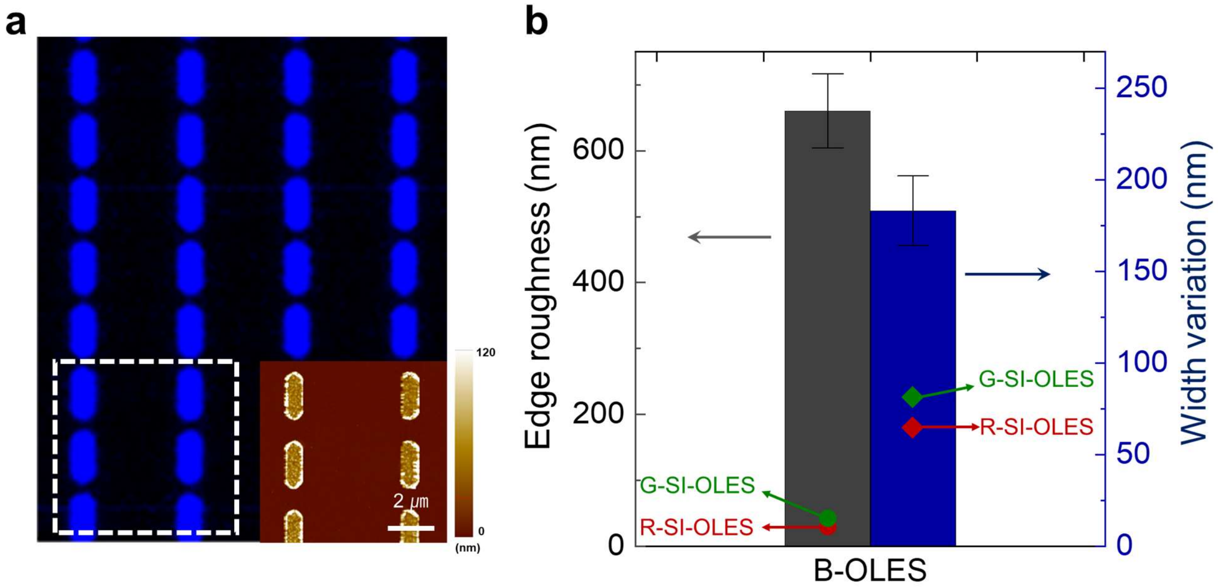

Supplementary Figure 20 | Characteristics of micropatterned B-OLES. **a**, Fluorescence and optical microscopic images of the B-OLES pattern array. The dimension of the micropatterns was 2 μm x 6 μm. **b**, Comparison of the pattern fidelity of the micropatterned B-OLES with that of the R- and G-SI-OLES. Mean values with ± standard variations are indicated in data.

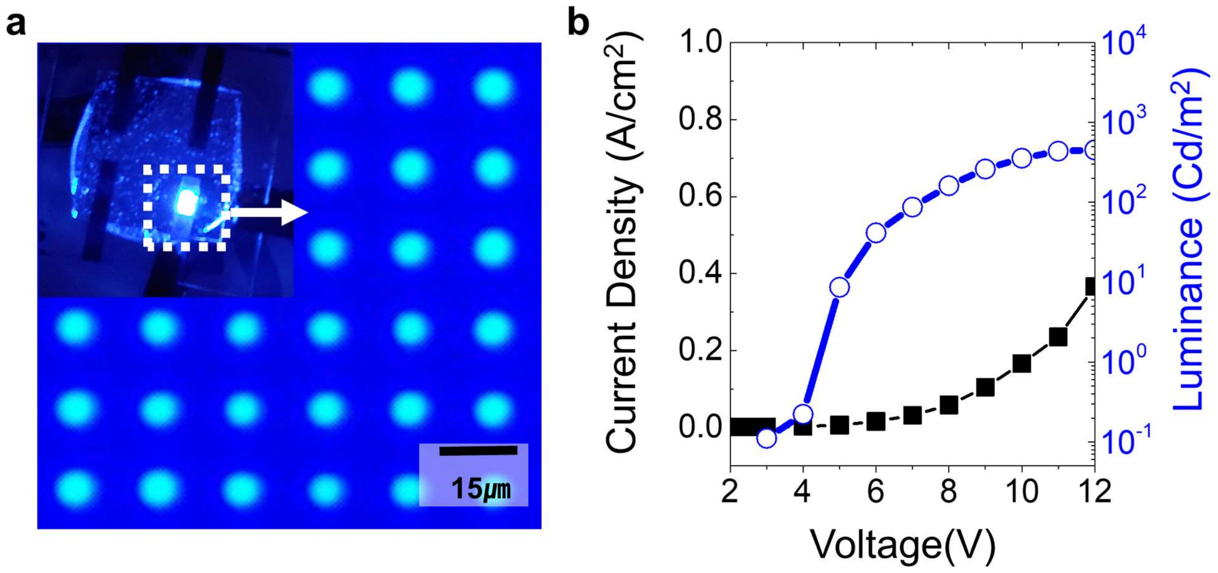

Supplementary Figure 21 | EL characteristics of pixelated B-OLED. a, Optical microscopy and photography images (inset) of electrically operating the B-OLED with 10 μm x 10 μm pixel size. **b**, J-V-L characteristics of the B-OLED.

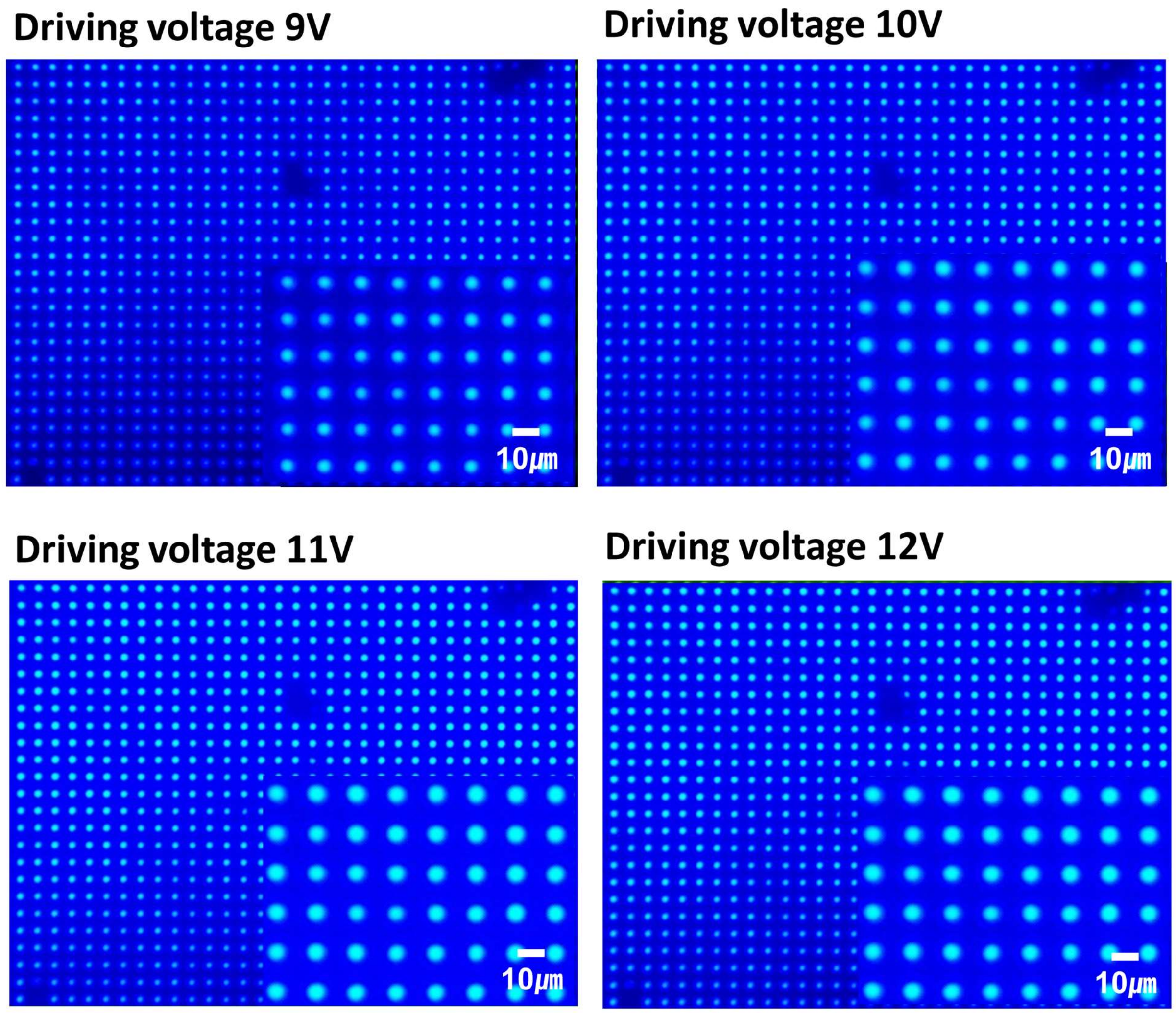

Supplementary Figure 22 | Voltage dependency of EL performance of the B-OLED. Operation image of B-OLED according to voltage variation based on dot pixels with 10 μm size.

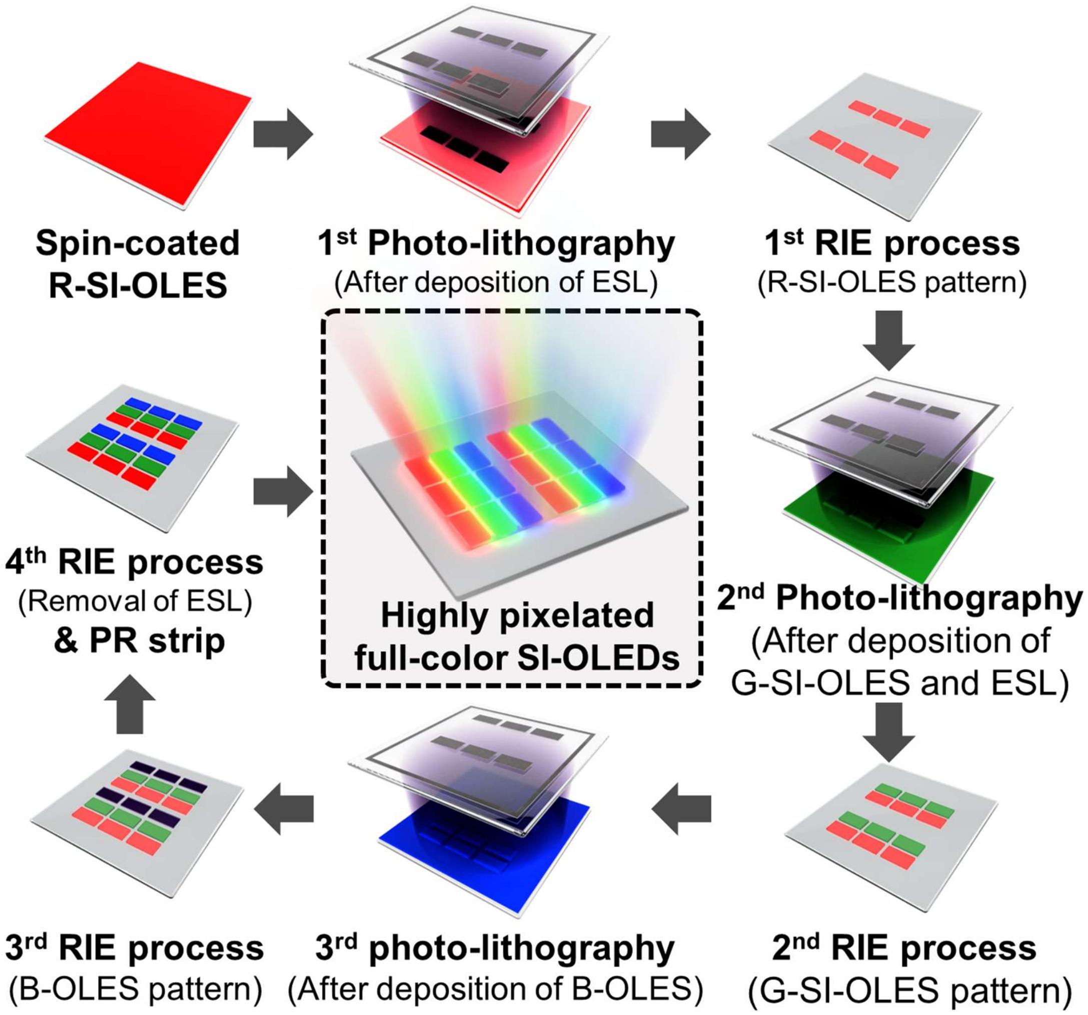

Supplementary Figure 23 | Fabrication process of the RGB micro-pixelation. Based on three cycles of consecutive reactive ion etching-coupled photolithography processes, highly pixelated RGB micropatterns can be achieved.

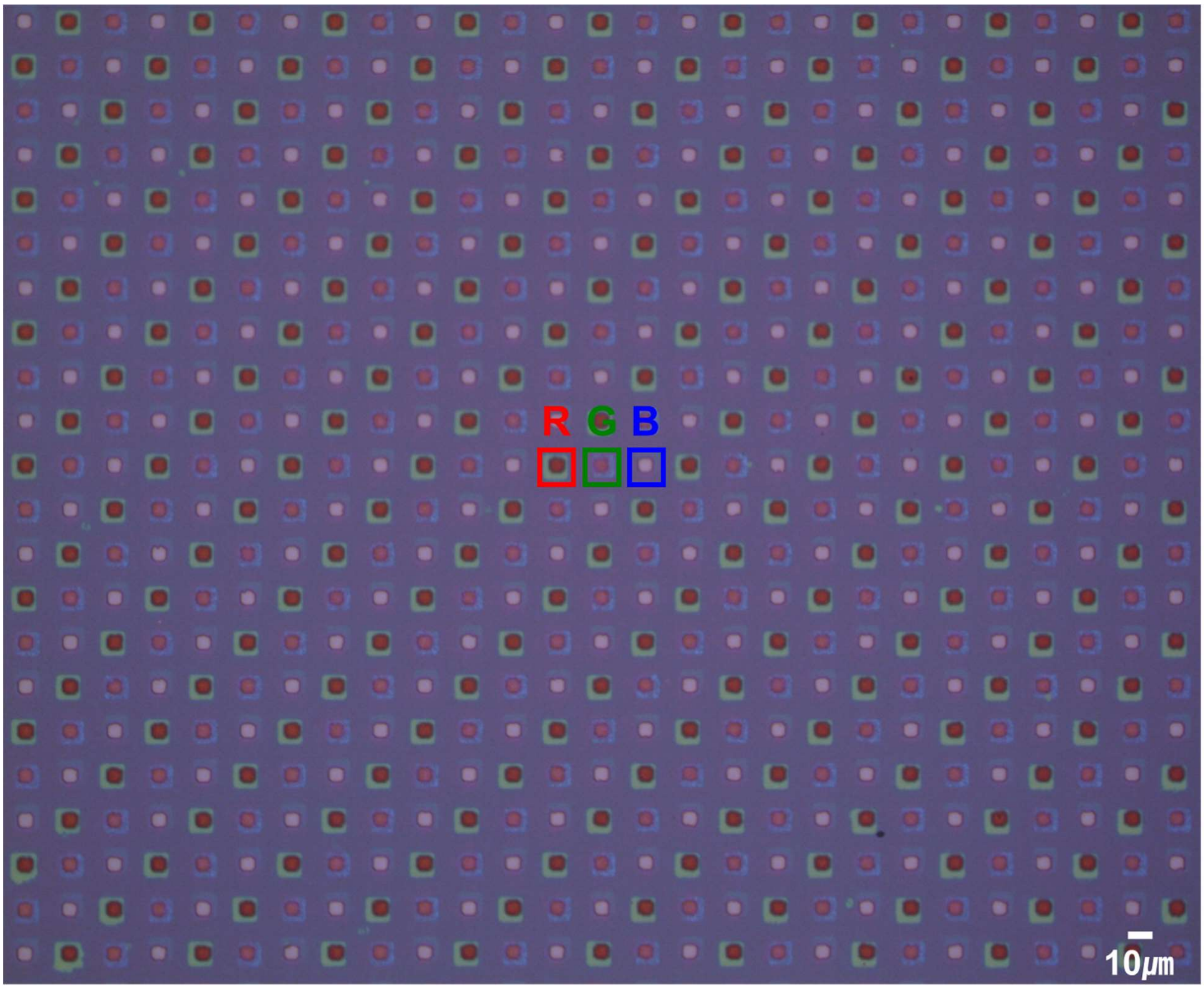

Supplementary Figure 24 | Optical microscope image of the full-color SI-OLED array. RGB pattern arrays corresponding to 949 ppi is fabricated by three cycles of consecutive RCP processes. The emission area (center part of pattern area) of each color is defined by the PDL layers.

Supplementary Table 1 | Micro-pattering performance of previously reported optical microlithography methods.

Organic semiconductor	Photo-crosslinking reactive group	Minimum pattern size	Minimum pitch size	Multi-patterning process	Ref
Super yellow	Azide	5 μm	10 μm	N/A	14
P(spirobifluorene-co-fluorenes)	Oxetane	125 μm	100 μm	Lateral pattern of Red/Green/Blue pixels	15
PEDOT:PSS<br>PTDPPTFT4	Vinyl<br>Carbene	2 μm	5 μm	Tandem structure of electrode/semiconductor/dielectric	16
PTDPPTFT4	Vinyl	1 μm	4 μm	N/A	17
PDPP4T<br>N2200	Carbene	5 μm	5 μm	Lateral and tandem pattern of *p*/*n*–type semiconductor	18
P(DPP2DT-TVT)<br>P(NDI3OT-Se2)	Azide	10 μm	10 μm	Tandem structure of electrode/semiconductor/dielectric	19
P(azido-styrene)-*co*-P(triphenylamine)	Azide	37 μm	42 μm	N/A	20
P3HT	Benzyl cation	15 μm	15 μm	N/A	21
P3HT	Vinyl	50 μm	100 μm	N/A	22
F8BT	Azide	100 μm	20 μm	N/A	23

Supplementary Table 2 | Summary of light-emitting performance of the OLEDs based on pristine OLES, SI-OLES, and micro-patterned SI-OLES.

Materials	Maximum luminance [cd/m^2]	Maximum current efficiency [cd/A]	Turn-on voltage [V]	EL peak wavelength [nm]
R-OLES	5270	0.28	2.03	584
R-SI-OLES	5619	0.28	2.05	588
Micro-patterned R-SI-OLES	2349	0.58	4.32	588
G-OLES	4940	0.42	2.54	544
G-SI-OLES	5303	0.46	3.01	544
Micro-patterned G-SI-OLES	3186	0.52	4.53	544

Supplementary References

1. Murawski, C. & Gather, M. C. Emerging biomedical applications of organic light-emitting diodes. *Adv. Opt. Mater.* **9**, 2100269 (2021).
2. Chang, C.-Y., Tsai, F.-Y., Jhuo, S.-J. & Chen, M.-J. Enhanced OLED performance upon photolithographic patterning by using an atomic-layer-deposited buffer layer. *Org. Electron.* **9**, 667-672 (2008).
3. Nabesawa, H. et al. Low-pressure plasma-etching of bulk polymer materials using gas mixture of CF_4 and O_2. *AIP Adv.* **3**, 112105 (2013).
4. Egitto, F. D. Plasma etching and modification of organic polymers. *Pure & Appl. Chem.* **62**, 1699-1708 (1990).
5. Arana, L. R. et al. Isotropic etching of silicon in fluorine gas for MEMS micromachining. *J. Micromech. Microeng.* **17**, 384-392 (2007).
6. Panduranga, P., Abdou, A., Ren, Z., Pedersen, R. H. & Nezhad, M. P. Isotropic silicon etch characteristics in a purely inductively coupled SF_6 plasma. *J. Vac. Sci. Technol. B* **37**, 061206 (2019).
7. Huang, B.-C. & Lin, Y.-J. Effect of the induced electron traps by oxygen plasma treatment on transfer characteristics of organic thin film transistors. *Appl. Phys. Lett.* **99**, 113301 (2011).
8. Qiu, Y. et al. Manipulating doping of organic semiconductors by reactive oxygen for field-effect transistors. *Phys. Status Solidi RRL* **12**, 1800297 (2018).
9. Jacobs, I. E. & Moule, A. J. Controlling molecular doping in organic semiconductors. *Adv. Mater.* **29**, 1703063 (2017).
10. Min, J.-H., Lee, G.-R., Lee, J.-K., Moon, S. H. & Kim, C.-K. Effect of sidewall properties on the bottom microtrench during SiO_2 etching in a CF_4 plasma. *J. Vac. Sci. Technol. B* **23**, 425 (2005).
11. Sugiura, H., Kondo, H., Tsutsumi, T., Ishikawa, K. & Hori, M. Effects of ion bombardment energy flux on chemical compositions and structures of hydrogenated amorphous carbon films grown by a radical-injection plasma-enhanced chemical vapor deposition. *C* **5**, 8 (2019).
12. Metzler, D., Bruce, R. L., Engelmann, S., Joseph, E. A. & Oehrlein, G. S. Fluorocarbon assisted atomic layer etching of SiO_2 using cyclic Ar/C_4F_8 plasma. *J. Vac. Sci. Technol. A* **32**, 020603 (2014).
13. Robey, S. W. & Oehrlein, G.S. Fluorination of the silicon dioxide surface during reactive ion and plasma etching in halocarbon plasmas. *Surf. Sci.* **210**, 429-448 (1989).

14. Jang, W. et al. Tetrabranched photo-crosslinker enables micrometer-scale patterning of light-emitting super yellow for high-resolution OLEDs. *ACS Photonics* **8**, 2519-2528 (2021).

15. Muller, C. D. et al. Multi-colour organic light-emitting displays by solution processing. *Nature* **421**, 829-833 (2003).

16. Zheng, Y.-Q. et al. Monolithic optical microlithography of high-density elastic circuits. *Science* **373**, 88–94 (2021).

17. Chen, R. et al. A comprehensive nano-interpenetrating semiconducting photoresist toward all-photolithography organic electronics. *Sci. Adv.* **7**, 1-10 (2021).

18. Wu, C. et al. An efficient diazirine-based four-armed cross-linker for photo-patterning of polymeric semiconductors. *Angew. Chem. Int. Ed.* **60**, 21521-21528 (2021).

19. Kim, M. J. et al. Universal three-dimensional crosslinker for all-photopatterned electronics. *Nat. Commun.* **11**, 1520 (2020).

20. Park, J. et al. Facile photo-crosslinking of azide-containing hole-transporting polymers for highly efficient, solution-processed, multilayer organic light emitting devices. *Adv. Funct. Mater.* **24**, 7588-7596 (2014).

21. Saito, Y., Sakai, Y., Higashihara, T. & Ueda, M. Direct patterning of poly(3-hexylthiophene) and its application to organic field-effect transistor. *RSC Adv.* **2**, 1285-1288 (2012).

22. Qiu, L. et al. Organic thin-film transistors with a photo-patternable semiconducting polymer blend. *J. Mater. Chem.* **21**, 15637 (2011).

23. Png, R. Q. et al. High-performance polymer semiconducting heterostructure devices by nitrene-mediated photocrosslinking of alkyl side chains. *Nat. Mater.* **9**, 152-158 (2010).
