# Supplementary material for: Silicone engineered anisotropic lithography for ultrahigh-density OLEDs
Source: Nat Commun. 2022 Dec 12;13:6775. doi: 10.1038/s41467-022-34531-y (PMC9744739; doi:10.1038/s41467-022-34531-y)
Supplement: Supplementary file 3 — Description of Additional Supplementary Files [file 41467_2022_34531_MOESM3_ESM.docx]

**Description of Additional Supplementary Files**

**File Name: Supplementary Movie 1
Description:** Operation of twocolor R/G-SI-OLED arrays. Electrically operating high-resolution R/G-SI-OLED arrays as presented Fig. 4a.
